# Appendiceal mucocele as an exceptional cause of ileocecocolic intussusception in adults: a case report

**DOI:** 10.1186/s13256-023-04133-3

**Published:** 2023-09-15

**Authors:** Aliou Zabeirou, Boubacar Efared, Lassey James Didier, Hama Younssa, Saidou Adama, Younoussa Moussa, Sani Rachid

**Affiliations:** 1 Department of General and Visceral Surgery, Hôpital Général de Référence, Niamey, Niger; 2 Faculty of Health Sciences, Abdou Moumouni University of Niamey, Niamey, Niger

**Keywords:** Ileocecocolic intussusception, Appendiceal mucocele, Mucinous cystadenoma, Laparoscopic right hemicolectomy

## Abstract

**Background:**

Intussusception is a rare condition in adults, accounting for 5% of intestinal intussusception and being responsible for approximately 1% of all adult bowel obstructions. Neoplastic origin is the most common etiology of intestinal intussusception in adults, unlike pediatric intussusception, which is usually idiopathic. Intussusception due to the appendiceal mucocele is exceptional, and only a few cases have been reported in the medical literature.

**Case presentation:**

We report the case of a 25-year-old black African male patient with no medical history. He presented to the emergency department for abdominal pain, nausea, and bilious vomiting. The abdominal examination revealed typical signs of acute bowel obstruction. Enhanced abdominopelvic computed tomography showed an invagination of the last ileal loop, cecum, and ascending colon into the lumen of the transverse colon, with a rounded image with hypodense content and some calcifications compatible with an appendiceal mucocele. An emergency exploratory laparoscopy was performed and confirmed the ileocecocolic intussusception. Right hemicolectomy and ileocolic anastomosis were performed. The patient recovery postoperatively was uneventful, and he was discharged 4 days later. Histological examination of the surgical specimen confirmed the diagnosis of mucinous cystadenoma.

**Conclusion:**

The symptoms of bowel intussusception with the appendiceal mucocele as the lead point in adults are similar to any other bowel intussusception. Differential diagnosis is often carried out thanks to the injected abdominal computed tomography scan.

## Introduction

Intussusception of the bowel is defined as an invagination of the intussusceptum (proximal bowel segment) into the intussuscipiens (adjacent bowel segment) that results in bowel obstruction [1]. Intussusception is a rare cause of bowel occlusion in adults, accounting for only 1–5% of all cases of intestinal occlusions [2]. The etiology of intussusception in adults is different from that in children. In adults, intussusception is more commonly associated with a benign or malignant lesion. Idiopathic intussusception accounts for 8% of all cases of intussusception in the adult population [3]. The diagnosis of intussusception in adults is more difficult because of the absence of specific signs and symptoms. Adult intussusception requires surgical intervention and bowel resection [4]. Bowel intussusception due to an appendiceal mucocele is an exceptional clinical presentation. Only a few cases have been reported in the medical literature [5].

We report the case of a 25-year-old male patient admitted for ileocecocolic intussusception due to appendiceal mucocele, with a laparoscopic right hemicolectomy and ileocolic anastomosis performed. The patient subsequently recovered uneventfully and was discharged on postoperative day 4.

## Case presentation

A 25-year-old black African male patient with no medical history presented to the emergency department for abdominal pain of 3-day duration. He also complained of bilious vomiting, nausea, and intermittent bowel subocclusion. Clinical examination found the patient hemodynamically stable, with blood pressure of 110/70 mmHg, pulse rate 75 beats per minute, respiration rate 18 breaths per minute, and body temperature of 37 °C. On physical examination, no pale conjunctiva or anicteric sclera were found. There was moderate abdominal distension and a firm mass in the right hypochondrium and epigastrium from abdominal palpation with diffuse abdominal tenderness.

Laboratory tests showed: a white blood cell count (WBC) of 6400/mm^3^, hemoglobin of 12.0 g/dL, platelet count of 263,000/mm^3^, prothrombin time (PT) 12.5 seconds [International Normalized Ratio (INR) 1.06], and blood chemistry was analyzed as blood urea nitrogen 27 mg/dL, creatinine 0.9 mg/dL, Na 139 mmol/dL, K 3.8 mmol/dL, Cl 102 mmol/dL, and C-reactive protein 24 mg/dL.

**Fig. 1:**
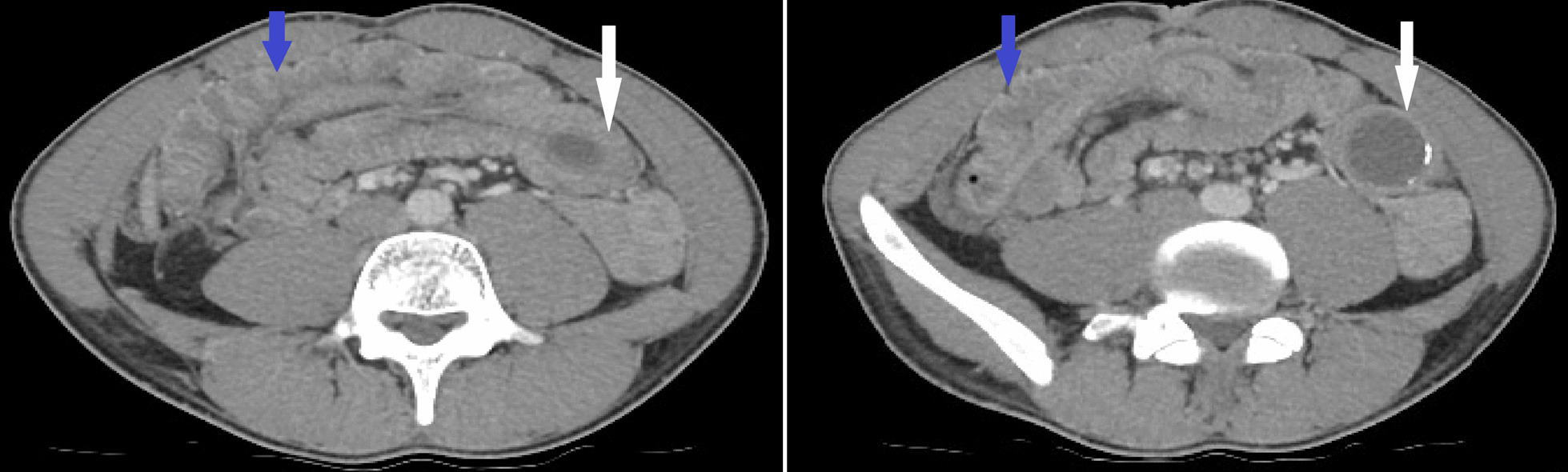
Axial CT scan demonstrated the ileocecocolic intussusception (blue arrows) and large appendiceal mucocele (white arrows)

**Fig. 2:**
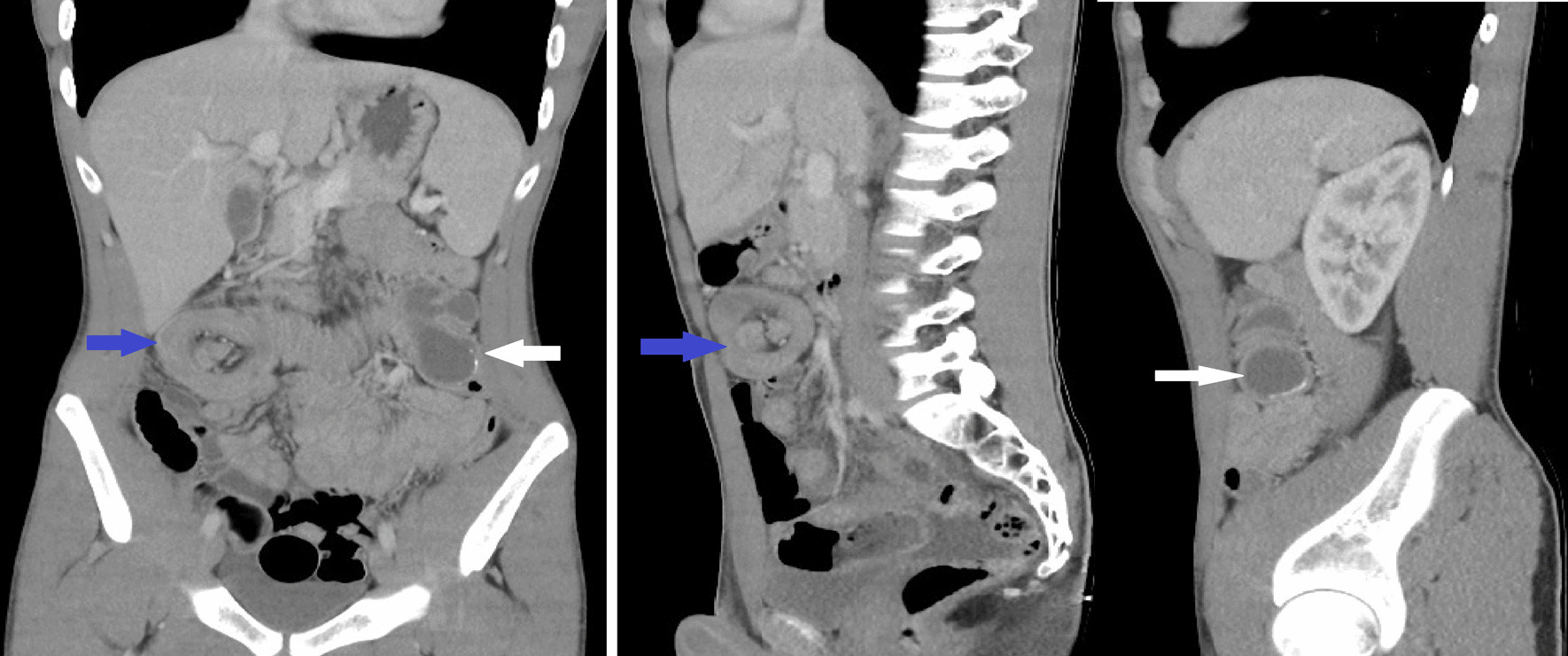
Frontal and longitudinal CT scan demonstrates the target or sausage-shaped lesions (blue arrows) and large appendiceal mucocele (white arrows)

An enhanced abdominopelvic computed tomography (CT) scan demonstrated an invagination of the last ileal loop, cecum, and the ascending colon into the lumen of the transverse colon as the target or sausage-shaped lesions. The intussusceptum (proximal bowel segment) also presented as a rounded image with hypodense content, and some calcifications located at the splenic angle were compatible with an appendiceal mucocele (Figs. 1, 2).

**Fig. 3:**
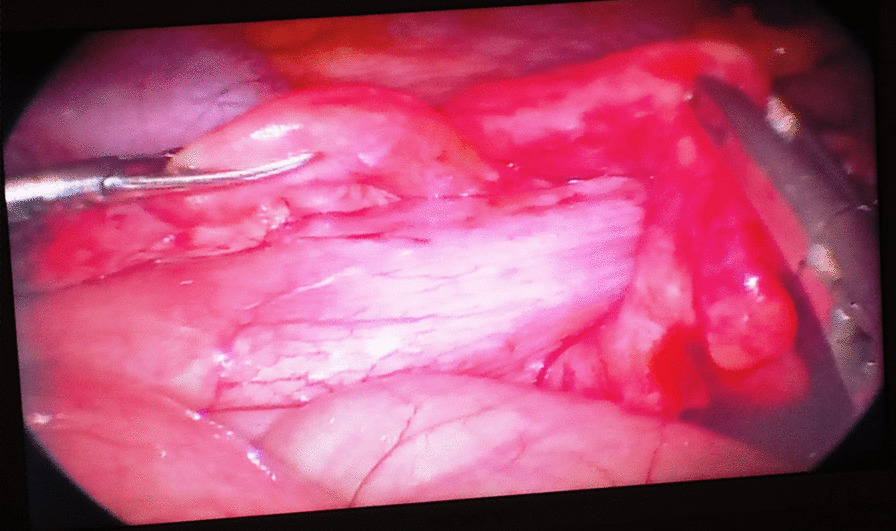
Intraoperative laparoscopic view showing ileocecocolic intussusception

**Fig. 4:**
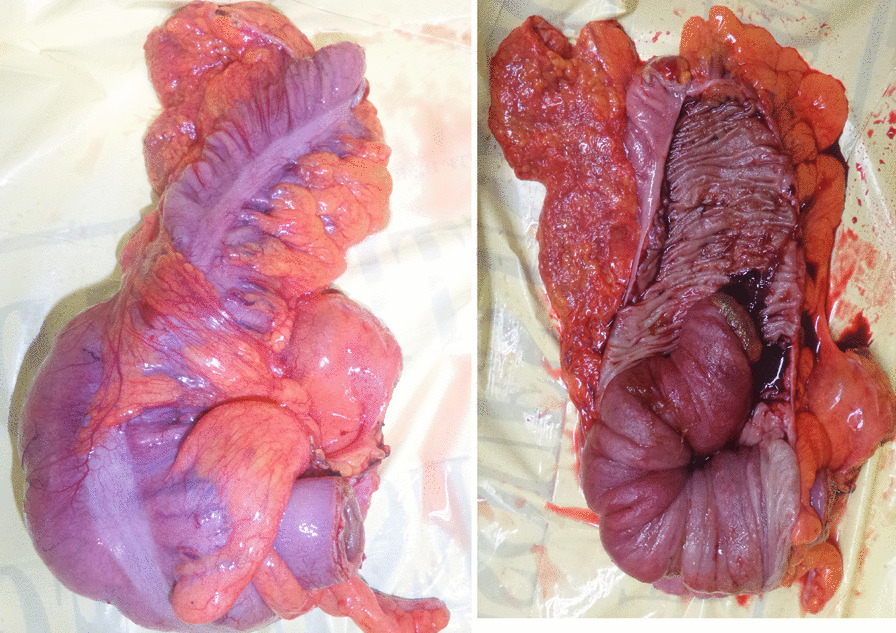
Postoperative specimen showing ileocecocolic intussusception

The patient underwent laparoscopy (Fig. 3); the ileocecocolic intussusception was confirmed and a right hemicolectomy and ileocolic anastomosis were performed (Fig. 4).

Postoperative recovery was uneventful, and the patient was discharged from the hospital 4 days later.

**Fig. 5:**
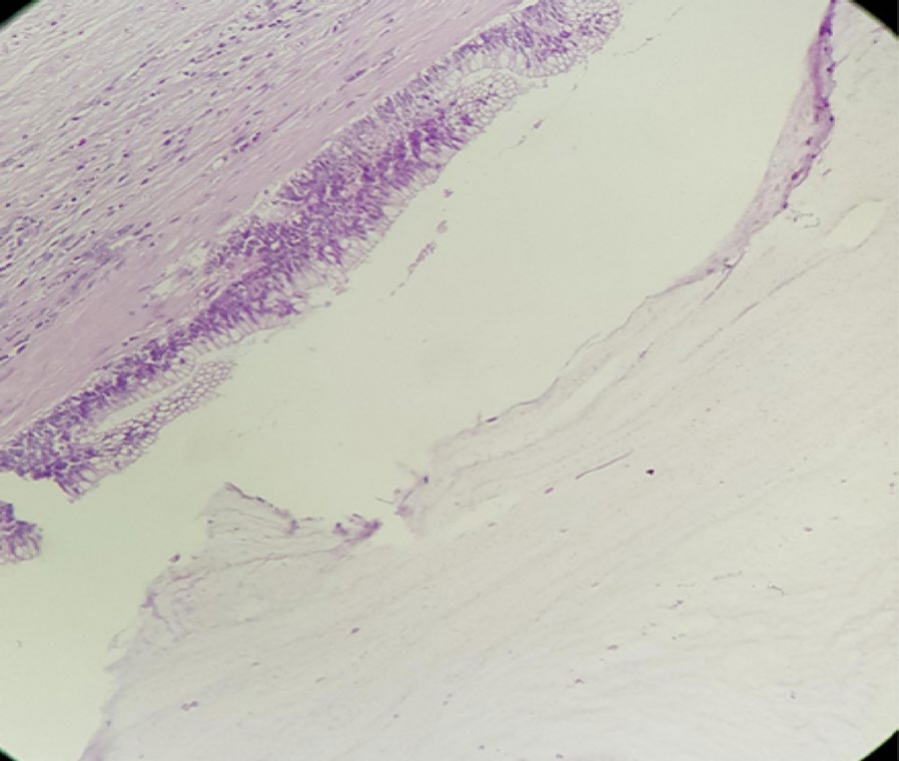
Histological image showing a cyst wall lined by mucinous pseudostratified epithelium [hematoxylin and eosin stain (HES) ×400]

Histopathological analysis of the surgical specimen revealed an intussusception with appendiceal mucinous cystadenoma (Fig. 5).

The patient has been under clinical and CT scan follow-up for 18 months with no intussusception recurrence or pseudomyxoma peritonei.

## Discussion

This case report is unique because it contributes to the medical literature regarding a massive intestinal intussusception whose intussusceptum consisted of the terminal ileum, the cecum, and the ascending colon; the whole of which was invaginated in the transverse colon, constituting the intussuscipiens. As a result, the appendiceal mucocele that constituted the lead point was located at the level of the left colic angle. The diagnosis was made by an abdominopelvic CT scan. A laparoscopic right hemicolectomy was performed, allowing for the rapid recovery of the patient. Histological examination of the surgical specimen had ascertained an intestinal intussusception due to a mucinous cystadenoma.

Intussusception is a common condition in children under the age of 3 years [6]. At this age, the etiology is usually idiopathic or secondary to a viral illness [7]. In adults, primary or idiopathic intussusception is rare, accounting for 8% of all cases [8]. More common intussusception in adults is secondary to a pathological lesion and results in a lead point, which can be benign (lymph node, mucosal polyp, appendix, lipoma, Meckel’s diverticulum) or malignant (adenocarcinoma, mucinous cystadenoma, lymphoma, gastrointestinal stromal tumor, carcinoid tumor) [9].

Appendiceal intussusception is secondary to abnormal peristaltic movements caused by a pathological appendix. This condition appears more frequently in adults (76%) than in children and is more common in women than in men, with a 2:1 ratio [10]. Intussusception of the appendix is an exceptional pathologic condition. In a study of 71,000 appendices, Collins reported that the incidence of this condition was 0.01% [11]. Fink *et al.* report that the etiologic factors of intussusception due to the appendix can be anatomical or pathological conditions [12].

The most frequent causes of appendiceal intussusception are endometriosis (33%), appendiceal mucocele (19%), and appendicitis (19%). In the remaining cases, the etiology is tumorous (carcinoid tumors, metastasis, hamartomas, or lymphomas) [13]. In 1941, Mc Swain [14] established an anatomical classification with five different types of appendiceal intussusception.

The clinical manifestations of appendiceal intussusception are varied, ranging from chronic colic, acute appendicitis syndrome, subocclusion, vomiting, or rectal bleeding to symptom-free [15]. The diagnosis of adult intussusception is very difficult because the classic triad of intermittent abdominal pain, jelly stools, and a palpable tender mass seen in children is rarely present in adults [16]. However, in adults, intussusception is usually detected using abdominal CT scans, colonoscopy, abdominal sonography, and magnetic resonance imaging [11, 13, 14]. The abdominal CT scan is the most commonly used diagnostic imaging technique for the diagnosis of appendiceal intussusception in adults. The CT scan shows “target” or “sausage”-shaped lesions and defines the nature, location, and relationship of the lesion to surrounding organs with accuracy that ranges from 60 to 100%.

Adult intussusception can lead to complications such as recurrence, bowel necrosis, bowel perforations, peritonitis, and sepsis. These complications occur when the diagnosis is missed or late, hence the need for prompt diagnosis and adequate treatment. The management of intussusception in adults is different from that in children. Surgical resection is indicated to remove the lead point [16]. Generally, intussusception has a good prognosis, and the important prognostic factor affecting the evolution of the condition is the etiologic lesion (malignancy) [17]. Mortality from adult intussusception accounts for 8.7% of cases of benign lesions and 52.4% of cases of malignant lesions [18].

## Conclusion

Intussusception due to appendiceal mucocele is an exceptional condition; it occurs with abdominal pain, subocclusion, and vomiting. These symptoms are not specific and lead to a late or missed diagnosis. The abdominal CT scan is the most commonly used diagnostic imaging technique for the diagnosis of appendiceal intussusception in adults. Laparoscopic management allows rapid recovery of the patient.

## Data Availability

All data generated or analyzed during this study are included in this published article.

## Abbreviations

WBC: White blood cells
PT: Prothrombin time
INR: International Normalized Ratio
Na: Blood sodium levels
K: Blood potassium levels
Cl: Blood chloride levels
CT: Computed tomography
